# Management of Respiratory Distress Syndrome due to COVID-19 infection

**DOI:** 10.1186/s12871-020-01095-7

**Published:** 2020-07-20

**Authors:** Jose R. Navas-Blanco, Roman Dudaryk

**Affiliations:** Department of Anesthesiology, Perioperative Medicine and Pain Management, University of Miami Miller School of Medicine, Jackson Memorial Hospital, 1800 NW 10 Avenue (M-820), Miami, FL 33136 USA

**Keywords:** Acute respiratory distress syndrome, Coronavirus disease 19, Severe acute respiratory syndrome coronavirus 2, Intensive care unit, Mechanical ventilation, Extracorporeal membrane oxygenation

## Abstract

The management of Acute Respiratory Distress Syndrome (ARDS) secondary to the novel Coronavirus Disease 2019 (COVID-19) proves to be challenging and controversial. Multiple studies have suggested the likelihood of an atypical pathophysiology to explain the spectrum of pulmonary and systemic manifestations caused by the virus. The principal paradox of COVID-19 pneumonia is the presence of severe hypoxemia with preserved pulmonary mechanics. Data derived from the experience of multiple centers around the world have demonstrated that initial clinical efforts should be focused into avoid intubation and mechanical ventilation in hypoxemic COVID-19 patients. On the other hand, COVID-19 patients progressing or presenting into frank ARDS with typical decreased pulmonary compliance, represents another clinical enigma to many clinicians, since routine therapeutic interventions for ARDS are still a subject of debate.

## Background

The novel Severe Acute Respiratory Syndrome Coronavirus 2 (SARS-CoV-2) infection, and its clinical manifestation as Coronavirus Disease 2019 (COVID-19) presents an unparalleled worldwide public health problem [1]. As reported on July 14, 2020, the pandemic of COVID-19 has already infected 12,964,809 patients and provoked 570,288 deaths (mortality of 4.39%) [2]. The disease introduces a unique pathophysiology and clinical course that puzzles the efficacy of the currently existing therapeutic approaches. This editorial presents an overview of the clinical experience gathered thus far from different centers around the world, and is not meant to constitute a guideline nor a standard of care for patients with COVID-19 pneumonia, given that the level of evidence behind the clinical approach to these patients is rapidly evolving.

### Pathophysiology of COVID-19

The proposed models of the underlying pathophysiology of COVID-19 associated acute respiratory distress syndrome (CARDS) are continuously investigated. Whether patients with CARDS present a similar pathophysiology for “typical” ARDS and match the Berlin Criteria definition remains a subject of debate. Based on current available clinical data the following components have been described in these patients:

- *Hypoxemic respiratory failure:* Direct cytopathic effects of the virus to the pneumocytes as opposed to inflammatory injury and virus-induced decrease in surfactant levels causing atelectasis are some of the unique pathologic findings seen in patients with COVID-19. Traditional diffuse alveolar hemorrhage and hyaline membrane formation have also been described [3]. Hypoxemia is the hallmark of the pulmonary derangement of the disease, in fact a case series of COVID-19 patients demonstrated the presence of significant hypoxemia with no signs of respiratory distress (*“silent hypoxemia”*) [4, 5].
  In light of these findings, Gattinoni et al. described two phenotypes of COVID-19 pneumonia: *Type 1 (*or *Type L, “non-ARDS”)*, a patient with normal to high pulmonary compliance (> 50 ml/cmH2O), low lung elastance, low lung weight and low lung recruitability. Hypoxemia is thought to be related to impaired blood flow and hypoxic pulmonary vasoconstriction, leading to a large ventilation/perfusion mismatch. *Type 2 (*or *Type H, “typical ARDS”)*, a patient with severe hypoxemia and markedly reduced pulmonary compliance (< 40 ml/cmH2O), high lung elastance, high lung weight (> 1.5Kg) and high lung recruitability. Overlapping characteristics of both these types of presentations may be present in a patient as an intermediate stage [5–8].
  However, a cohort study by Ziehr et al. presented 66 intubated patients with COVID-19 with similar pulmonary mechanics of “typical” ARDS, based on decreased lung compliance, dead space and response to prone positioning [9]. Therefore, further research is required to determine the description of CARDS as an “atypical” presentation of ARDS.
- *Cytokine Storm:* Dysregulated and excessive immune responses may lead to significant systemic damage. Mononuclear cells such as neutrophils and monocytes in the patient’s lung tissues and peripheral blood produce elevated levels of pro-inflammatory cytokines such as interleukin-6 (IL-6), interleukin-1 and tumor necrosis factors [10]. Evidence supports a marked cytokine release in COVID-19 infection, directly related to the severity and mortality of the disease, and this is reflected by high levels of interleukin-6 (IL-6), ferritin and C-reactive Protein (CRP) [11, 12].
- *COVID-19-related Hypercoagulability:* A distinct prothrombotic state as opposed to a consumptive coagulopathy has been described in COVID-19 patients, secondary to a markedly increased levels of fibrin and fibrinogen. This mechanism is synergistic with the cytokine storm and the virus-induced endothelial dysfunction. Consequently, serum levels of D-dimer are a strong prognostic factor of poor outcomes [12–14].

## Initial approach to patients with COVID-19 pneumonia

### Clinical presentation and initial laboratory findings

Fever has been reported with variable incidence among case series, ranging between 43 and 98% of the patients, hence the absence of fever does not exclude COVID-19 infection [15–17]. Cough is the second most common symptom (59–72%), followed by myalgia (15%) and fatigue (23%). Gastrointestinal symptoms (diarrhea, nausea and vomiting) has been reported in 10% of the patients usually preceding the onset of fever and dyspnea [17]. ARDS has been described to be present in 17–41% of these patients, with a median time from first symptom to progression to ARDS of 8 days [4, 17, 18].

In regard to initial laboratory findings, white blood cell count tends to be normal and lymphopenia is present in 80% of the patients [19]. CRP has been found to be consistently elevated in patients with COVID-19 infection and shares an inverse relationship with oxygen requirement levels, and may be used for mortality prognostication [20, 21]. Other prognostic laboratory values sharing a direct relationship with mortality in COVID-19 infection are IL-6, LDH and serum ferritin and D-dimer [22]. Procalcitonin levels are moderately elevated in these patients and have not been consistently associated to predict mortality of the disease [19]. In addition to the reverse-transcription polymerase chain reaction (RT-PCR) for COVID-19, routine blood cultures and viral panel should be sent to rule out the presence of any other microorganism as a main culprit or as co-infection [23].

### Imaging findings

Chest radiograph demonstrates the typical patchy ground glass opacities, which tends to be towards the periphery and basal fields of the lungs, and its sensitivity has been reported around 59% [19, 24]. In regards to Computerized Tomography (CT) findings, its sensitivity seems to be related to the symptomatology of the patient at the moment the test is performed: 86–97% in patients with respiratory symptoms and 50% in patients with constitutional symptoms only [19, 25, 26].

Zhou et al. in a cohort of 100 COVID-19 patients who underwent 272 CT of the chest, demonstrated a predominant peripheral distribution (62%), followed by a combined peripheral plus central distribution (38%). The vast majority had a bilateral lung compromise (92%), and a significantly involvement of the middle, lower and posterior zones [27]. Not all patients suspected for COVID-19 pneumonia require CT imaging. The decision of requesting this exam should be made in a case-by-case basis based on the clinician’s judgement, given the potential risk for pathogen dissemination during the patient transportation and the consequent exposure to the healthcare personnel.

Lung ultrasound in patients with COVID-19 pneumonia has also been described and its findings appear to correlate satisfactorily with the CT findings. Peng et al. described the presence of scattered B-lines which tends to coalesce as the disease progresses. Thickening of the pleural line as well as pleural effusions have been described [28].

### Nosocomial spread and Aerosolization

Environmental control is paramount to be considered in the initial approach to patients with COVID-19 pneumonia. Droplet precautions have been advocated by multiple organizations. The World Health Organization and the Australian and New Zealand Intensive Care Society recommend airborne precautions when aerosol-generating procedures are expected in COVID-19 patients, including: face mask ventilation, non-invasive ventilation, endotracheal intubation, open airway suctioning, aerosolized medications, bronchoscopy, disconnection of the patient from the ventilator and cardiopulmonary resuscitation. Despite the high risk of contamination during these procedures, current evidence suggests that meticulous use of personal protective equipment is effective to prevent infection among healthcare personnel [29–31].

## Approach to the non-intubated COVID-19 patient

Monitoring respiratory drive and effort are essential in spontaneously breathing patients to detect early stages of respiratory fatigue. Although non-invasive ventilatory (NIV) maneuvers have been employed in patients with COVID-19 pneumonia and the literature regarding the adequate timing for intubation is controversial, most authors agree that in the presence of impaired respiratory mechanics, worsening of respiratory acidosis and most importantly decreased mental status, endotracheal intubation and mechanical ventilation should not be delayed [7, 32].

For spontaneously breathing patients with mild-to-moderate dyspnea and hypoxemia, non-responsive to regular low-flow nasal cannula, initial approach may involve the use of high flow nasal cannula (HFNC) and awake prone positioning based on limited clinical data. HFNC, although initially controversial due to its aerosolizing potential, it was found to be safe in further studies, with a bio-aerosol dispersion not significantly different from regular nasal prongs [33]. Airborne precautions among the treating staff should be maintained and the patient should be placed in a negative pressure room if available [29, 30, 34]. Yang et al. demonstrated higher survival among patients with HFNC when compared to other means of mechanical ventilation, either non-invasive or invasive [35]. Awake prone positioning involves the patient lying on his/her abdomen as a method to improve secretion clearance and recruitment of atelectatic lung tissue at the dependent lung bases [34].

NIV support (continuous positive airway pressure [CPAP] or Bi-level positive airway pressure [BiPAP]) has been employed in COVID-19 patients as a last resource to circumvent endotracheal intubation after failing HFNC and awake prone positioning, although no formal recommendation regarding their use has been released due to the risk of aerosolization [29]. CPAP seems to provide the greatest advantage among NIV for COVID-19 pneumonia patients, since it provides the greatest amount of mean airway pressure leading a more effective alveolar recruitment when compared with BiPAP. This latter has been found to be more appropriate for specific certain group of patients with other concomitant co-morbidities (i.e. chronic obstructive pulmonary disease, congestive heart failure) [7]. Due to the aerosolizing potential of the traditional NIV techniques, the use of a “helmet CPAP” as a means to non-invasive ventilation interface have been proposed [36]. The use of bronchodilators should be minimized and instead nebulization metered dose inhalers should be employed [30].

It is paramount to closely monitor the respiratory efforts in COVID-19 patients spontaneously breathing (regardless of the use of HFNC or NIV support). In the context of early CARDS, a high respiratory drive may lead to the generation of high transpulmonary pressures and lung stress with consequent increased risk to develop the so-called “patient self-inflicted lung injury” (P-SILI) [8, 37].

## Approach to the intubated COVID-19 patient

Institution of endotracheal intubation and mechanical ventilation should be made as soon as possible regardless of the phenotype of the COVID-19 pneumonia, when signs of respiratory distress are associated to the severe hypoxemia. The following ventilatory strategies represent an expert opinion, based on current and rapidly evolving evidence in patients with CARDS, therefore further data is required to confirm the efficacy of these maneuvers.

### Management of Mechanical Ventilation

Gattinoni et al. proposed tailored modifications to the usual ARDS principles based on the phenotype of the COVID-19 pneumonia. In intubated type 1 phenotype patients, the management of hypoxemia should be directed to improve the ventilation/perfusion mismatch by liberalized tidal volumes (7-8 ml/kg ideal body weight, to avoid resorption atelectasis), limited PEEP levels (8-10cmH2O) and keeping the respiratory rate < 20 breaths per minute [6, 7, 32].

As lung damage progresses, type 2 phenotype arises following a similar pattern of a “typical” ARDS (bilateral infiltrates, decreased respiratory system compliance and increased lung weight) [6]. The standard approach of lung protective ventilation through low tidal volumes (6 ml/kg ideal body weight), PEEP levels (<10cmH2O), FiO2 levels as tolerated to avoid poor tissue perfusion, plateau pressures <30cmH2O, lower driving pressures (target of 13–15 cmH2O) and permissive hypercapnia have consistently demonstrated to be an accepted intervention for CARDS [38]. The aim is to avoid ventilator-induced lung injury by reducing lung and vascular stress [32]. While some authors advocate for high levels of PEEP (<15cmH2O) in phenotype 2 patients, based on the high lung elastance and increased non-aerated lung tissue [8], a single-center observational study demonstrated poor lung recruitability in spite of high PEEP levels. Lung recruitment was more evident after prone positioning in the population analyzed [39].

Airway pressure release ventilation (APRV) may be considered early in the course of intubated patients with moderate to severe ARDS, in order to provide adequate alveolar recruitment. The application of APRV continues to be limited, given that many providers are not familiar with this ventilation mode or its titration methodology [40]. No data has been published yet regarding APRV utility in patients with CARDS.

### Role of prone positioning, neuromuscular blockade and corticosteroids

Prone positioning leads to a relieve of severe hypoxemia due to reduction of overinflated lung areas, promoting of alveolar recruitment and decreasing ventilation/perfusion mismatch [39]. The Proning Severe ARDS Patients (PROSEVA) trial, performed by Guerin et al. demonstrated a significant decrease in 28-day and 90-day mortality in patients with severe ARDS [41]. Prone positioning has been advocated in intubated patients with CARDS. Pan et al. demonstrated increased lung recruitability and PaO2/FiO2 improvement after prone positioning in patients with moderate CARDS [39]. The main obstacle continues to be its implementation and generalization among each institution. Trained and qualified nursing and respiratory therapy staff is the most important factor to obtain successful results, as severe life-threatening events may occur at any given time (self-extubation, hemodynamic instability, lack of adequate sedation, pressure ulcers) [29, 30].

In regards to neuromuscular blockade, in an effort to avoid ventilator dyssynchrony, adjust tidal volumes and decrease airway resistance with the goal to decrease lung parenchyma inflammation, neuromuscular blocking agents were introduced as part of the alternative therapies for severe ARDS [42]. No formal clinical trial or body of evidence has been published yet regarding the use of neuromuscular blockade in patients with CARDS. The Surviving Sepsis Campaign recommends the use of intermittent doses of neuromuscular blocking agents to facilitate lung protective ventilation in COVID-19 patients [29].

In regards to inflammatory attenuators for patients with CARDS, the RECOVERY trial, a large multicenter, randomized controlled trial, demonstrated that patients in the intervention group (receiving Dexamethasone 6 mg for 10 days, either enterally or intravenously) reduced death by up to one-third in hospitalized patients with severe respiratory complications from COVID-19. The effect seemed to be more prominent in patients on mechanical ventilation (number needed to treat: 8) and intermediate in those who required supplemental oxygen only (number needed to treat: 25). The trial did not show any benefit from Dexamethasone in patients who did not required respiratory support [43].

### Role of pulmonary vasodilator therapies

Selective pulmonary vasodilation is thought to improve ARDS secondary to redistribution of blood from poorly ventilated areas to those with higher ventilation, thereby decreasing the shunt fraction and correcting hypoxemia. *Nitric Oxide* and *Prostaglandins* (e.g. PGI_2_ [epoprostenol]), despite its pulmonary vasodilatory properties have failed to demonstrate a mortality benefit in ARDS [44]. Nitric Oxide use during the COVID-19 patients is controversial [45]. Some authors advocate for its potential anti-viral activity following the results of a study performed during the SARS-CoV outbreak in 2004 [46]. Currently there is no recommendation for the use of pulmonary vasodilators in patients with ARDS due to COVID-19, other than a last resource (rescue therapy) for refractory hypoxemia [29].

### Role of extracorporeal membrane oxygenation (ECMO)

When refractory hypoxemia ensues and alternative therapies fail, the use of extracorporeal maneuvers becomes appropriate. Despite that the use of ECMO has increased substantially in the past decades, its use still remains controversial. Two large multi-center RCT have offered a contradictory view regarding the use of ECMO in ARDS. CESAR trial (*Conventional ventilatory support* versus *ECMO for severe ARDS*) performed by Peek et al. (2009), introduced encouraging results demonstrating a significant improvement in survival without severe disability at 6 months. The authors of this trial concluded that for patients with severe ARDS with potentially reversible causes not responsive to conventional management ECMO should be instituted. On the other hand, the EOLIA trial (ECMO to Rescue Lung Injury in ARDS) performed by Combes et al. (2018), concluded that 60-day mortality was not significantly lower within patients in the ECMO group and therefore the trial was stopped given pre-specified rules, although a post-hoc bayesian analysis of this trial demonstrated a posterior probability of mortality benefit from ECMO in the population analyzed with very severe ARDS [47, 48].

In light of these controversial results and in the midst of a worldwide pandemic caused by the COVID-19, with multiple patients expected to develop severe ARDS, the Extracorporeal Life Support Organization (ELSO) emitted a consensus guideline for the use of ECMO in these patients. The ELSO emphasizes that center experience as well as provider and team training are the most prominent factors to determine the use of extracorporeal techniques in patients with COVID-19. In regards to patient prioritization, the consensus guideline recommends highest priority to younger patients with minor or no co-morbidities as well as healthcare providers, highlighting that patients with poor prognosis, advanced age with multiple co-morbidities, do-not-resuscitate status, terminal disease or severe central nervous system damage should be excluded.

In order to determine patient eligibility, the ELSO consensus on CARDS provides a forthright algorithm centered in providing conservative management (prone positioning, neuromuscular blockade, pulmonary vasodilators, high PEEP, recruitment maneuvers) for patients with PaO2/FiO2 ratio < 150 mmHg. If the patient develops worsening refractory hypoxemia (PaO2/FiO2 ratio < 80 mmHg for > 6 h, *or* PaO2/FiO2 ratio < 50 mmHg for > 3 h) or signs of poor tissue perfusion and hypercarbia (pH < 7.25 with partial pressure of arterial carbon dioxide [PaCO2] > 60 mmHg), then the patient should be considered for ECMO, assuming no contraindications are present. Also, for patients with PaO2/FiO2 ratio > 150 mmHg, but with signs of poor tissue perfusion and hypercarbia, ECMO should be considered as well [49].

## Conclusion

The management of ARDS secondary to COVID-19 infection poses significant clinical, logistical and ethical dilemmas. Hypoxemia itself does not constitute an indication for intubation if pulmonary mechanics are preserved. On the contrary, prolonged breathing efforts either spontaneous or assisted with NIV, while mental status deteriorates and respiratory acidosis develops are detrimental. Efforts should be directed to identify those patients with significant respiratory distress who require intubation and mechanical ventilation as delay in these interventions may be associate to poor outcomes. Establishing a tailored institutional protocol for the clinical approach in these patients while maintaining provider safety is paramount.

## Data Availability

Non-applicable.

## Abbreviations

SARS-CoV-2: Severe Acute Respiratory Syndrome Coronavirus 2
COVID-19: Coronavirus Disease 2019
ARDS: Acute Respiratory Distress Syndrome
CARDS: COVID-19 Acute Respiratory Distress Syndrome
IL-6: Interleukin-6
CRP: C-Reactive Protein
LDH: Lactate Dehydrogenase
RT-PCR: Reverse-Transcription Polymerase Chain Reaction
PaO2: Partial Pressure of Arterial Oxygen
FiO2: Fraction of Inspired Oxygen
PEEP: Positive End-Expiratory Pressure
CT: Computerized Tomography
HFNC: High-Flow Nasal Cannula
NIV: Non-invasive Ventilation
CPAP: Continuous Positive Airway Pressure
BiPAP: Bi-level Positive Airway Pressure
P-SILI: Patient Self-Inflicted Lung Injury
RCT: Randomized Controlled Trial
ICU: Intensive Care Unit
APRV: Airway Pressure Release Ventilation
ECMO: Extracorporeal Membrane Oxygenation
ELSO: Extracorporeal Life Support Organization
PaCO2: Partial Pressure of Arterial Carbon Dioxide
